# Implementation of Automated Blood Culture With Quality Assurance in a Resource-Limited Setting

**DOI:** 10.3389/fmed.2021.627513

**Published:** 2021-05-21

**Authors:** Anja von Laer, Micheline Ahou N'Guessan, Fidèle Sounan Touré, Kathrin Nowak, Karin Groeschner, Ralf Ignatius, Johannes Friesen, Sara Tomczyk, Fabian H. Leendertz, Tim Eckmanns, Chantal Akoua-Koffi

**Affiliations:** ^1^Postgraduate Training for Applied Epidemiology, Department of Infectious Disease Epidemiology, Robert Koch Institute, Berlin, Germany; ^2^Department of Infectious Disease Epidemiology, Robert Koch Institute, Berlin, Germany; ^3^European Programme for Intervention Epidemiology Training, European Centre for Disease Prevention and Control, Stockholm, Sweden; ^4^Laboratoire Central, Centre Hospitalier et Universitaire de Bouaké, Bouaké, Côte d'Ivoire; ^5^Department of Microbiology, Medical Care Unit Labor 28 GmbH, Berlin, Germany; ^6^Research Group Epidemiology of Highly Pathogenic Microorganisms, Robert Koch Institute, Berlin, Germany

**Keywords:** blood culture, sub-Saharan Africa, bacterial infection, laboratory automation, quality control

## Abstract

**Background:** Blood cultures (BC) have a high clinical relevance and are a priority specimen for surveillance of antimicrobial resistance. Manual BC are still most frequently used in resource-limited settings. Data on automated BC performance in Africa are scarce. We implemented automated BC at a surveillance site of the *African Network for improved Diagnostics, Epidemiology and Management of Common Infectious Agents* (ANDEMIA).

**Methods:** Between June 2017 and January 2018, pairs of automated BC (BacT/ALERT^®^FA Plus) and manual BC (brain-heart infusion broth) were compared at a University hospital in Bouaké, Côte d'Ivoire. BC were inoculated each with a target blood volume of 10 ml from the same venipuncture. Automated BC were incubated for up to 5 days, manual BC for up to 10 days. Terminal subcultures were performed for manual BC only. The two systems were compared regarding yield, contamination, and turnaround time. For quality assurance, isolates were retested in a German routine microbiological laboratory.

**Results:** BC sampling was increased from on average 24 BC to 63 BC per month. A total of 337 matched pairs of BC were included. Automated BC was positive in 36.5%, manual BC in 24.0% (*p*-value < 0.01), proportion of contamination was 47.9 and 43.8%, respectively (*p*-value = 1.0). Turnaround time of positive BC was shortened by 2.5 days with automated compared to manual BC (*p* < 0.01). Most common detected pathogens in both systems were *Klebsiella* spp. (26.0%) and *Staphylococcus aureus* (18.2%). Most contaminants were members of the skin flora. Retesting of 162 isolates was concordant in 79.6% on family level.

**Conclusions:** Implementing automated BC in a resource-limited setting is possible and improves microbiological diagnostic performance. Automated BC increased yield and shortened turnaround times. Regular training and mentorship of clinicians has to be intensified to increase number and quality of BC. Pre-analytical training to improve diagnostic stewardship is essential when implementing a new microbiological method. Retesting highlighted that manual identification and antimicrobial susceptibility testing can be of good quality and sustainable. The implementation of automated tools should be decided individually according to economic considerations, number of samples, stable supply chain of consumables, and technical sustainability.

## Introduction

In low- and middle-income countries (LMIC), infectious diseases remain one of the main causes of morbidity and mortality. Febrile illness is a leading cause for admission to hospitals in Africa (1). Despite the major burden of infectious diseases, the availability of diagnostic microbiology services for bloodstream infections other than malaria is often limited by cost, infrastructure, and personnel constraints. Antimicrobial resistance (AMR) compromises the outcome of bloodstream infections and reduces treatment options. Although reliable data on AMR in Africa is scarce according to the World Health Organization (WHO), the available data indicate that AMR is increasing in Africa (2). Furthermore, second-line drugs to treat infections with resistant bacteria are not easily accessible in all countries. One of the five objectives of the WHO Global Action Plan on AMR is to strengthen the knowledge and evidence base through surveillance and research (3). WHO defines blood cultures (BC) as a priority specimen for AMR surveillance and it is recommended to prioritize key clinical specimens in resource-limited settings (4, 5). WHO also highlights diagnostic stewardship as an integral part to build up AMR surveillance systems, defining this as the “coordinated guidance and interventions to improve appropriate use of microbiological diagnostics to guide therapeutic decisions” (6).

During the last two decades, automated systems with computer-driven monitor of CO_2_ concentration for the detection of microorganisms in BC have been developed. Comparisons of automated BC systems to conventional manual procedures have shown a higher sensitivity and a shorter time to positivity (7–9). As resources are limited, manual BC is often used in African countries (10, 11). Data on automated BC implementation in resource-limited settings, especially in sub-Saharan Africa, are scarce (11, 12).

To combat infectious diseases including AMR, a surveillance system has been developed at sentinel sites in Côte d'Ivoire among other West African countries as part of the African Network for improved Diagnostics, Epidemiology and Management of Common Infectious Agents (ANDEMIA, https://www.andemia.org) (13).

Bouaké, the second largest city in Côte d'Ivoire, is located in the central part of the country 350 kilometers north of Abidjan, the economic and political capital and has a population of ~700,000 inhabitants. Bouaké was the center of military and political crises in Côte d'Ivoire, particularly from 1999 to 2011. The University Hospital Bouaké (CHU-B), the country's only academic hospital outside Abidjan, had to suspend all except the most basic medical services between 2002 and 2011. CHU-B today has 268 beds, 27,189 admissions and 62,515 consultations in 21 departments per year.

In 2012–2014, a study on manual BC from severely ill patients at CHU-B showed a positivity rate of 22.5% with highest rates in pediatrics (14). The most commonly isolated pathogen was *Klebsiella pneumoniae*, followed by *Salmonella enterica*. In 2017, a study on the resistance patterns of *Klebsiella pneumoniae* from clinical samples at CHU-B showed that most isolates derived from BC and that 84% produced extended spectrum beta-lactamases (ESBL) (15). Routine BC sampling might be hampered by shortage of resources and empiric antibiotic treatment is often started without diagnostics. In 2016, on average 24 BC were processed per month (16). To improve diagnostics and to support culture-guided therapy, an automated BC system was implemented at CHU-B in the frame of the ANDEMIA project.

The aim of the present study was to verify automated BC in the laboratory, to compare automated BC with manual BC regarding proportion positive, proportion contaminated and turnaround-times and to improve BC sampling in order to improve patient care as well as AMR surveillance.

## Methods

We conducted a prospective laboratory study from 28 June 2017 to 18 January 2018 to implement automated BC in a resource-limited setting. The implementation was accompanied by a 6-week on-site mentorship period of a clinical microbiologist from Germany and 1 week of theoretical and practical training on BC processing and antimicrobial susceptibility testing (AST) with an international team of experts from Burkina Faso, Côte d'Ivoire, the Democratic Republic of Congo and Germany.

### Participants

BC from consecutive patients admitted to any department at CHU-B with self-reported fever or fever on admission (≥38.0°C) and suspected bloodstream infection were included in the study. No exclusion criteria were applied. The decision to take blood samples for culture rested solely on the physicians' clinical judgement. The study was imbedded in the routine workup.

### Training of Clinicians

The departments were informed about the study by the head of the laboratory on 26 June 2017. Clinicians were instructed to take a set of BC for automated and manual processing from each patient above 2 years of age with an indication for BC sampling. Training of clinicians on good practice for BC sampling was performed in a one-hour session by laboratory staff in the three departments that have sent most BC. One-page job aids on how to take a set of BC for children and adults were distributed to each department in the hospital.

### Blood Culture Processing

Automated BC were processed using the BacT/ALERT^®^ 3D system (bioMérieux, Marcy L'Etoile, France). Commercially produced BacT/ALERT^®^FA bottles were used for adults above 15 years of age and BacT/ALERT^®^ PF bottles were used for children below 15 years of age. In children when insufficient blood volume was obtained and who were below three years of age, only the BacT/ALERT^®^ PF bottle was inoculated. For manual BC, commercially available BC was used for adults and children (HIMEDIA HiCombiTM Dual Performance Medium and HIMEDIA BHI Broth – Supplemented x/0.05% SPS, HIMEDIA Laboratories, Mumbai, India). In case of stock-out, self-prepared brain-heart infusion (BHI) broth (60 ml for adult and 30 ml for pediatric bottles) in suitable glass bottles was used instead. Commercially available BC media had to be used as suitable glass bottles to be filled manually were not sufficiently available for the increased number of samples. Clinicians were asked to take 20 ml blood from adults and 10 ml from children above 2 years via syringe, and to fill a pair of BC bottles (i.e., 10 ml each in adults and 3–4 and 5–6 ml for children for automated and manual BC, respectively, according to manufacturer's instructions). As weighing each bottle before and after blood sampling to measure the exact volume of inoculated blood was not feasible in the daily routine and would have delayed BC processing, different types of BC bottles were weighed beforehand. Each BC bottle was weighed after processed in the laboratory to calculate the inoculated blood volume.

Both BC systems were processed according to the newly revised Standard Operating Procedures (SOPs) in the laboratory. Automated BC bottles were incubated for up to 5 days according to manufacturer's instructions. Manual BC bottles were incubated at 35 ± 2°C for up to 10 days with final subculture and checked daily for turbidity or other signs of growth (e.g., haemolysis, pellicle formation on surface or gas production). Positive BC were inoculated onto locally produced 5% columbia blood agar, chocolate agar and Hektoen enteric agar, dependent on the result of the Gram stain, and incubated at 35 ± 2°C in ambient or CO_2_-enriched air (candle jar) for 24–48 h (Bio-Rad Laboratories Ltd., Hercules, United States of America and Oxoid Ltd., Hampshire, United Kingdom). Identification of isolates was done by cultural morphology, biochemical and antigenic methods (Supplementary Figure 1). Gram stain was performed from positive BC and from colonies on agar. Antimicrobial susceptibility testing (AST) was done for isolated pathogenic bacteria by Kirby-Bauer disc diffusion test using references from the European Committee on Antimicrobial Susceptibility Testing (EUCAST) (17). If no breakpoint for disc diffusion was available for EUCAST (e.g., vancomycin for staphylococci), the reference from the Clinical & Laboratory Standards Institute (CLSI M100, 31st edition) was used instead.

### Data Collection

The routine laboratory request form for microbiological investigation for BC provided to clinicians was adapted to include information on demographics, time of sampling and antibiotic treatment. The attending microbiologist defined the respective recovered isolate as contaminant or pathogen individually based on multiple criteria including identification (e.g., common skin contaminant), number of positive BC and clinical information. A positive BC was defined as a BC with subsequent growth of microorganisms.

The proportion of positive BC was defined as the number of positive BC divided by the number of all processed BC.

The proportion of contaminated BC was defined as the number of contaminated positive BC divided by the number of positive BC.

The proportion of BC with a false-positive signal was defined as the number of BC that had a positive signal without growth in subculture divided by the number of BC with that respective information.

The turnaround-time was measured as the days from BC sampling until the final microbiological result was available in the laboratory.

The time-to-detection was measured as the hours from loading the BC bottle into the machine or incubator until the BC was flagged positive.

The time-to-analysis was measured as the hours from loading the BC bottle into the machine or incubator until the positive BC bottle was removed and processed.

The time-to-positivity was measured as the hours from BC sampling until the BC was flagged positive.

### Quality Assurance/Retesting of Isolates

Since CHU-B did not have an active external quality assurance programme, an inter-laboratory comparison was done and isolates were retested in a German routine microbiological laboratory.

Bacterial and fungal isolates, irrespective if they were isolated from automated and/or manual BC, were stored in ESwabs (Copan, Brescia, Italy) at 4°C until the end of the study and then shipped to Berlin, Germany. They were revived on blood agar and checked for purity. If multiple strains were isolated from one BC, all strains were retested, accordingly. Microorganisms were identified using matrix-assisted laser desorption ionization time-of-flight mass spectrometry (MALDI-TOF MS, Bruker Daltonik, Bremen, Germany; software: MBT 7854 MSP Library, 2018) or Vitek^®^2 (bioMérieux, Marcy L'Etoile, France; software version: 8.01, 2018). AST was performed using Vitek^®^2 or disc diffusion using references from EUCAST (18). Isolates identified as *Salmonella* Typhi at CHU-B were excluded from retesting in Germany for safety reasons as handling is recommended under higher safety levels. Comparison of identification of isolates was done stepwise regarding family/group, genus, and species level. If species level could not be determined using conventional methods (e.g., for non-fermenters or coagulase-negative staphylococci), genus or group level was counted. Family or group were defined as follows: Non-fermenter, *Enterobacterales*, gram-positive rods (*Bacillus* spp.), *Micrococcaceae/Staphylococcus* spp., fungi, *Enterococcus* spp./*Streptococcus* spp. Genus was defined as follows: Non-fermenter (other than *Pseudomonas* spp.), *Escherichia* spp., *Alcaligenes* spp., *Bacillus* spp., *Citrobacter* spp., *Enterobacter* spp., *Enterococcus* spp., *Klebsiella* spp., yeasts, *Micrococcus* spp., *Pseudomonas* spp., *Salmonella* spp., *Staphylococcus* spp., *Streptococcus* spp., *Stenotrophomonas* spp. and *Kocuria* spp.

### Data Analysis

All data were double-entered into the EpiData software (EpiData Association, Odense, Denmark) (19). Analyses were conducted using Stata version 15.1 (StataCorp, Texas, USA) and Microsoft Excel (2010). Automated and manual BC were compared in terms of proportion positive, proportion contaminated, turnaround-times, recovery of different bacteria and maintenance. If automated or manual BC bottles were received in the laboratory only with no matching pair, they were excluded from the comparison. Statistical differences between automated and manual BC were calculated using McNemars exact test, to compare medians the Wilcoxon signed-rank test was used. A *p*-value below 0.05 was defined as statistically significant. As different manual BC bottles had to be used, a subgroup analysis of proportion positive and contaminated in different bottles was performed using Fisher's exact test. Interpretations of AST from CHU-B and Germany were compared. If duplicates from automated and manual BC were re-tested, only one isolate was included in the analysis. If the interpretation of AST for the same species was different in automated and manual BC, the more resistant test was included in the analysis.

### Ethics

The studies involving human participants were reviewed and approved by Charité University Ethics Committee Berlin, Germany (reference number: EA2/230/17) and the National Ethics Committee for Research in Abidjan, Côte d'Ivoire (reference number: 141/MHSP/CNER-km). Isolates were obtained through the hospitals routine diagnostic and used for this study.

## Results

### Comparison of Automated and Manual BC

In total, 440 BC were sent for analysis during the study period of 7 months. BC sampling was increased from on average 24 BC to 63 BC per month. Of those, 103 automated BC arrived without corresponding manual BC in the laboratory (90 pediatric and 13 adult BC) and were subsequently removed from the final analysis. In total, 337 pairs of automated and manual BC were included in the analysis. The median age of patients was 31 years (range: 0–88 years). Of 333 patients with information on sex, 53.8% of patients were male (*n* = 179). Of 330 patients with information on antibiotic treatment, 191 (57.9%) had received antibiotics before BC sampling with a median of 3 days (range: 0–21 days). The three most common administered antibiotics were ceftriaxone, amoxicillin and clavulanic acid, and gentamycin.

Volumes of BC bottles varied, but were similar in automated and manual BC. Median volume in adult BC was 10.0 ml and in pediatric BC 3.6 and 3.0 ml in automated and manual BC, respectively.

Of 337 pairs, the results of 259 (76.9%) manual and automated BC were concordant (Table 1). Of those, 196 were negative (75.7%), whereas 63 were positive (24.3%). In 78 discordant pairs (23.1%), 18 BC showed growth only in manual BC (23.1%) and 60 only in automated BC (76.9%).

**Table 1:** Summary of concordant and discordant pairs of automated BC and manual BC, CHU-B, Côte d'Ivoire 2017–2018, *N* = 337.

**Results Manual BC**	**Results Automated BC**		
	**Negative**	**Positive**	**Total**
Negative, *n* (%)	196 (58.2%)	60 (17.8%)	256 (76.0%)
Positive, *n* (%)	18 (5.3%)	63 (18.7%)	81 (24.0%)
Total, *n* (%)	214 (63.5%)	123 (36.5%)	337 (100%)

The proportion of positive BC was significantly higher in automated BC (*n* = 123, 36.5%) than in manual BC (*n* = 81, 24.0%, *p* < 0.01, Table 2). The proportion of contaminated BC was not significantly higher in automated BC (*n* = 58, 47.5%) than in manual BC (*n* = 35, 43.2%, *p* = 1.0). The proportion of BC that show growth with a pathogen was 23.3% in automated BC and 15.2% in manual BC.

**Table 2:** Comparison of proportion positive and contaminated and turnaround-times with responding 95% Confidence Intervals (95% CI] of automated BC and manual BC, CHU-B, Côte d'Ivoire 2017–2018, *N* = 337.

**Variables**	**Automated BC**			**Manual BC**			**Difference**	***p*-value**
	***n***	**Value**	**(95% CI)**	***n***	**Value**	**(95% CI)**		
Proportion **positive** in %	123	36.5	(31.3–41.9)	81	24.0	(19.6–29.0)	12.5	<0.01
Proportion **contaminated** in %	58	47.9	(38.8–57.2)	35	43.8	(32.7–55.3)	4.1	1.0
median **TAT^1^** (turnaround-time) in days	332	5.0	(5.0–5.0)	331	11.0	(11.0–12.0)	6.0	<0.01
median **TTP^2^** (time-to-positivity) in hours	112	19.7	(17.6–22.1)	69	69.2	(45.3–113.6)	49.5	<0.01
median **TTD^3^** (time-to-detection) in hours	126	17.0	(15.6–19.2)	80	90.4	(59.0–11.7)	73.4	<0.01
median **TTA^4^** (time-to-analysis) in hours	127	26.8	(25.2–28.0)	79	96.4	(69.2–117.0)	69.6	<0.01

1 *TAT: BC sampling until final result*.

2 *TTP: BC sampling until flagged positive*.

3 *TTD: Loading in BC machine/incubator until flagged positive*.

4 *TTA: Loading in BC machine/incubator until removal and initial work*.

Subgroup analysis of different types of manual BC bottles (commercially available vs. self-produced BC) regarding proportion positive and proportion contaminated did not show a significant difference (data not shown).

Automated BC showed significantly less frequently a false positive signal: whereas 40.2% (*n* = 88) of manual BC signaled positive, but no microorganisms grew in sub-culture, 5.6% (*n* = 10, *p* < 0.01) of automated BC showed a false positive signal. The median inoculated blood volume in these false positive BC was 10 ml.

Turnaround times were shorter with automated BC than with manual BC irrespective of the result of the BC (*p* < 0.01, Table 2). The turnaround time from BC sampling until final results was 6.0 days shorter with automated BC than with manual BC. The time from loading the BC bottle into the machine or incubator until the positive BC was removed and analysis was started was shortened by 3 days (69.6 h) with automated BC compared to manual BC. For positive BC the turnaround time from BC sampling until final result was shortened with automated BC by 2.5 days and the time from incubating the BC bottle until the start of analysis by 76.4 h (data not shown).

**Figure 1:**
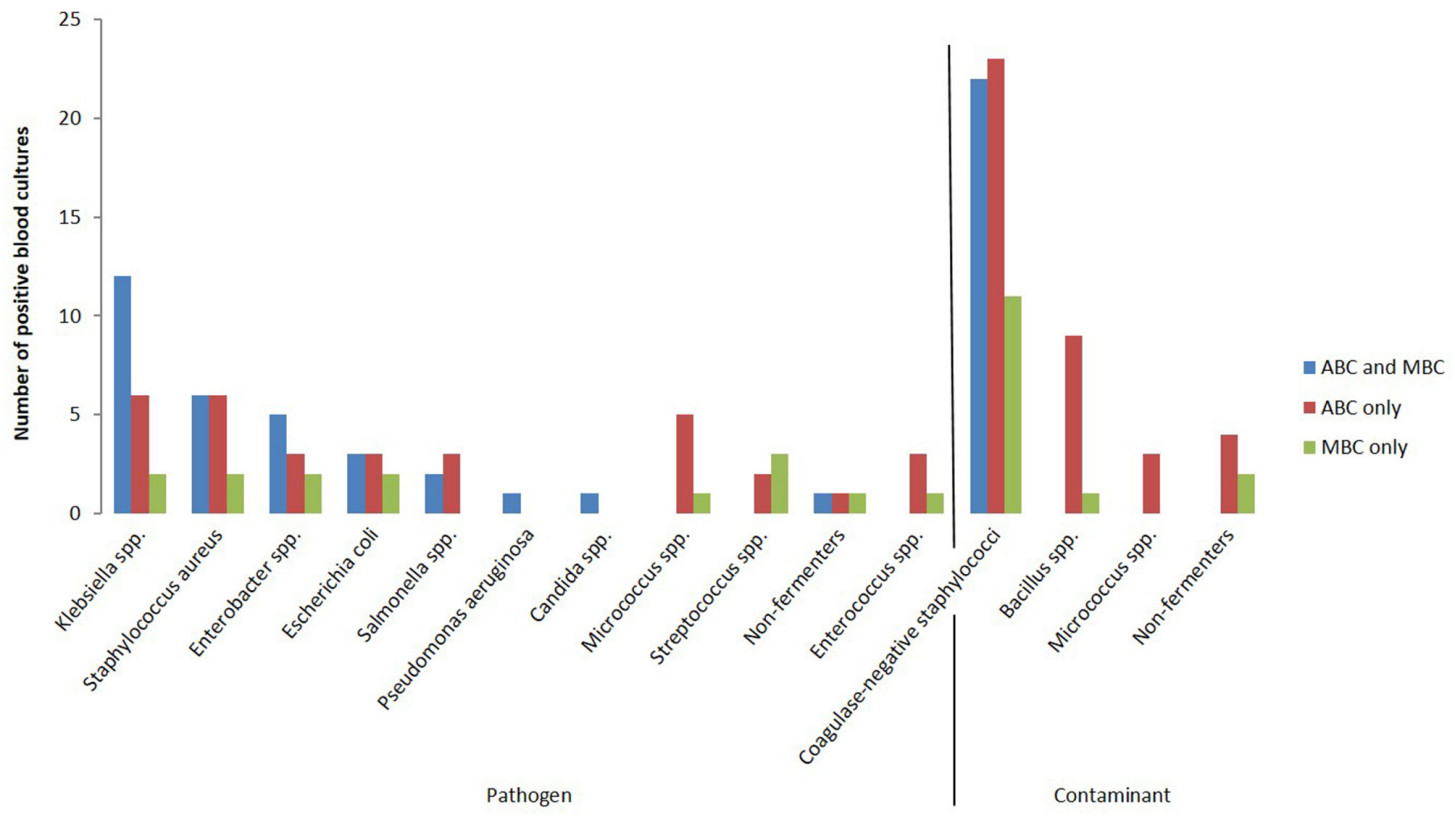
Detected bacteria with automated BC (ABC) and manual BC (MBC), CHU-B, Côte d'Ivoire 2017–2018, *N* = 337.

The most often detected bacteria in automated and manual BC were coagulase-negative staphylococci (*n* = 56/152, 36.8%); all of those were interpreted as contaminants (Figure 1). The most often detected pathogens were *Klebsiella* spp. (*n* = 20/152, 13.2%), *Staphyloccoccus aureus* (*n* = 14/152, 9.2%) and *Enterobacter* spp. (*n* = 10/152, 6.6%). The detection rate was higher with automated BC for almost all detected bacteria.

**Figure 2:**
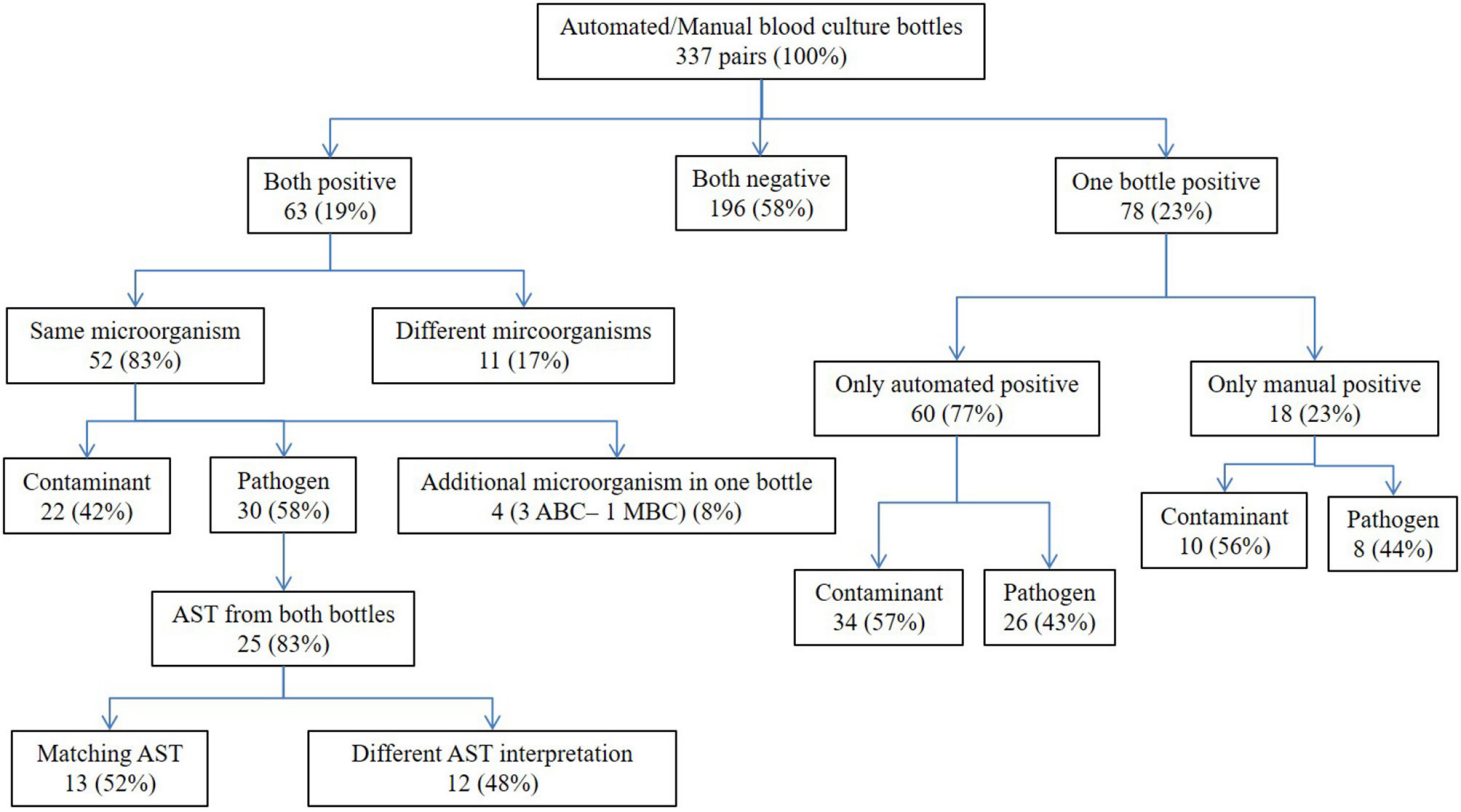
Comparison of proportion positive, detected microorganism and antimicrobial susceptibility testing (AST) results for automated BC (ABC) and manual BC (MBC), CHU-B, Côte d'Ivoire 2017–2018, *N* = 337.

If automated BC and manual BC were both positive (*n* = 63), the same microorganism was identified in 82.5% (*n* = 52, Figure 2). Of those, 30 microorganisms (57.7%) were interpreted as pathogens. For 83.3% of those pathogens (*n* = 25), results of antimicrobial susceptibility testing (AST) were available from both, automated and manual BC including 19 *Enterobacterales* and six *Staphylococcus aureus* isolates. Clinical interpretation of AST results in automated and manual BC were concordant in 52.0% (*n* = 13); in 48.0% (*n* = 12), the AST of at least one antibiotic was interpreted differently in automated and manual BC for the same pathogen.

The subgroup analysis of discordant pairs, where the automated BC was positive and the final result of the corresponding manual BC was negative, revealed that in 6.7% (4/60), the manual BC was false-positive, i.e., the BC was signaled positive, but no microorganisms were detected in Gram stain or subculture. In 11.1% (2/18) of discordant pairs in which the final result of the manual BC was positive but the automated BC was negative, the automated BC bottle was false-positive. In discordant pairs, the median inoculated blood volume was higher in manual BC than in automated BC. Adult automated BC bottles were filled with a median of 9.5 ml and manual BC with 10.0 ml; pediatric automated BC bottles were filled with a median of 3.2 ml and manual BC with 9.4 ml.

Initial and maintenance requirements for the automated and commercially available manual BC systems varied (Table 3). In particular, the hands-on time was reduced with automated BC compared to manual BC. The expenses for BC bottles were also similar for automated and commercially available manual BC as we could not establish a reliable delivery chain for suitable glass bottles for self-prepared BHI broth for the increased request of BC.

**Table 3:** Comparison of initial and maintenance requirements for automated and manual BC, CHU-B, Côte d'Ivoire 2017–2018.

	**Automated BC**	**Manual BC**
Costs of machine	• Initial funding necessary	• Incubator was available
Costs of BC bottles	• Similar if commercially available manual BC bottles are used	
Training	• Intensive training and continuous mentorship of all laboratory staff required • Delayed incubation if staff during weekend or night shifts are not trained in loading the machine	• Staff were already trained in manual BC • Additional training on ventilation of bottles • Incubation of BC bottles could be started throughout the weekend and night
Maintenance	• Technical support from manufacturer was assured • Reliable delivery of bottles was assured • Expert with training in troubleshooting was available in the laboratory	• Delivery of bottles was not always reliable • No sustainable delivery for suitable glass bottles for self-prepared BHI broth could be established
Hands-on time	• Reduced, no daily manual inspection needed • Incubation shortened to 5 days without final sub-culture • False-positive signals were reduced (10/178, 5.6%)	• Daily inspection for growth • Incubation for 10 days, final sub-culture recommended • Often false-positive signals without subsequent growth of microorganisms (88/219, 40.2%)

### External Quality Control - Retesting of Isolates

**Figure 3:**
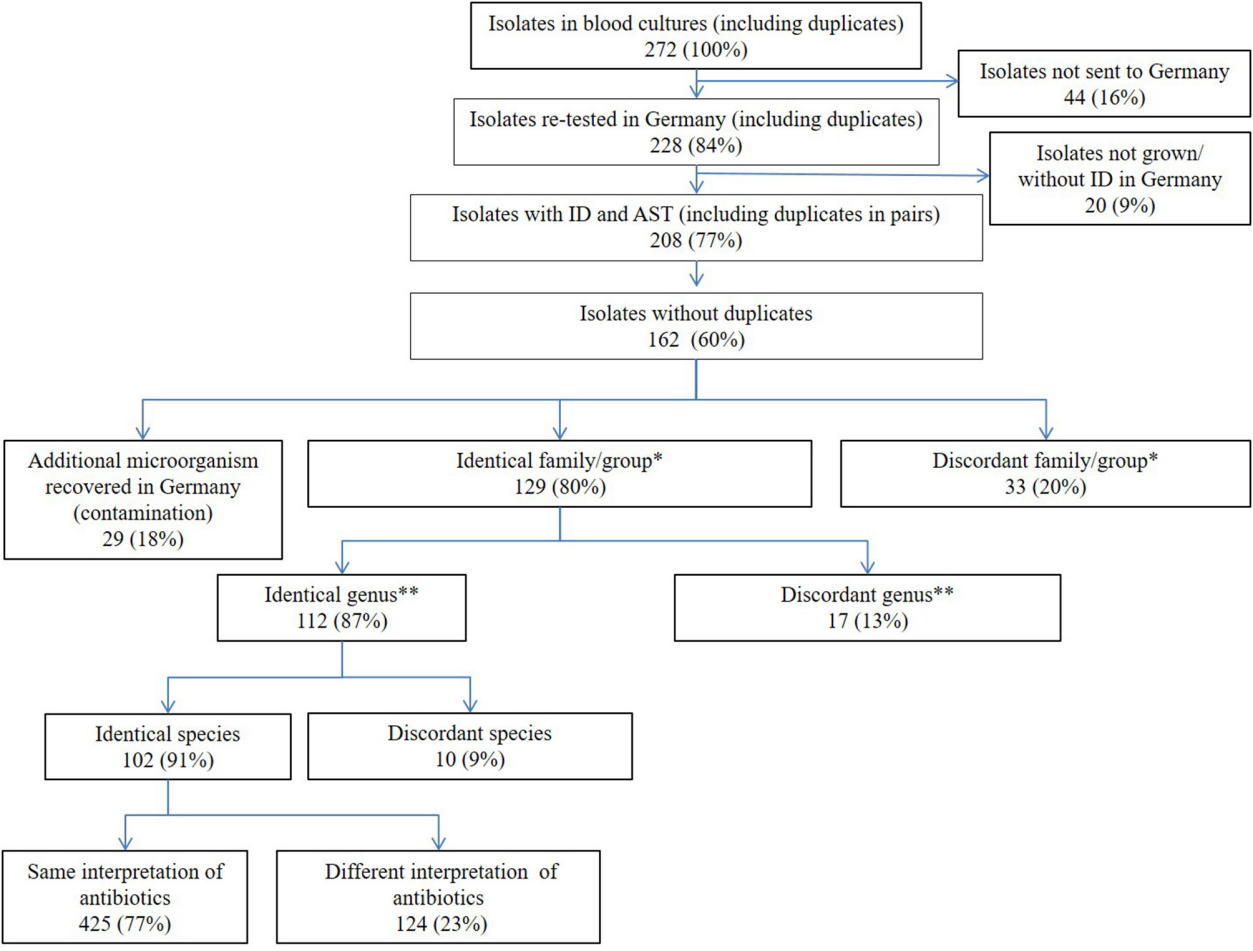
Retesting of isolates from blood cultures in German laboratory for quality assurance, Côte d'Ivoire and Germany 2017–2018, *n* = 272. *Family or group were defined as follows: Nonfermenter, *Enterobacterales*, gram-positive rods (*Bacillus* spp.), *Micrococcaceae/Staphylococcus* spp., fungi, *Enterococcus* spp./*Streptococcus* spp. **Genus was defined as follows: Nonfermenter (other than *Pseudomonas* spp.), *Escherichia* spp., *Alcaligenes* spp., *Bacillus* spp., *Citrobacter* spp., *Enterobacter* spp., *Enterococcus* spp., *Klebsiella* spp., yeasts, *Micrococcus* spp., *Pseudomonas* spp., *Salmonella* spp., *Staphylococcus* spp., *Streptococcus* spp., *Stenotrophomonas* spp. and *Kocuria* spp.

Of all 440 BC (including single automated and manual BC), 269 were positive (61.1%). Of those 269 positive BC, 272 microorganisms could be recovered as in some BC multiple bacteria were found. However, not all isolates could be stored. Thus, in total, 228 isolates with identification at CHU-B were sent for retesting to Germany including duplicates recovered in corresponding automated and manual BC (Figure 3). Of those, 20 isolates could not be revived or identified. Identification using MALDI-TOF had to be repeated 45-times (36 isolates); in 6 cases, VITEK-2 was used for identification, but one isolate that had been identified as *Klebsiella* spp. could not be identified with automated identification tools (MALDI-TOF and VITEK^®^). Isolates that had been identified as *Salmonella* Typhi were excluded from retesting (*n* = 1). In total, 208 isolates (77% of all isolates sent for retesting) could be retested with identification and AST. Excluding duplicates from automated and manual BC pairs, 162 unique isolates were retested in Germany. In 29 cases, more microorganisms were found in the sample in Germany than detected at CHU-B. Of those 162 isolates, identification on family or group level was concordant in 79.6% (*n* = 129). Differences were mostly found in isolates identified as *Staphylococcus* spp. at CHU-B (*n* = 16/62, 25.8%). They were identified as non-fermenters (8/16, 50.0%) or *Bacillus* spp. (5/16, 31.3%) in retesting. In 112 isolates (69.1%), the identification was concordant on genus level and in 102 isolates (63.0%) concordant on species level. Of all *Staphylococcus* spp. identified at CHU-B, 67.7% (*n* = 42/62) were concordant on species level in retesting, whereas 59.7% (*n* = 40/67) of *Enterobacterales* were concordant on species level. Of those 102 isolates, AST interpretation was concordant in 77.4% of antibiotics (*n* = 425) in retesting. Teicoplanin was concordant in 10% and vancomycin in 40% of tests. Here, the AST at CHU-B was more often non-susceptible than the AST in retesting. Eight of 10 (80.0%) *Staphylococcus aureus* isolates with concordant species identification in retesting were concordant in methicillin-susceptibility or –resistance testing (MSSA or MRSA). One MSSA was identified as MRSA and one MRSA was identified as MSSA in retesting. Of 42 *Enterobacterales* isolates, 83.3% (*n* = 35) were concordant in susceptibility to third-generation cephalosporins (cefotaxime, ceftriaxone or ceftazidime). Susceptibility to carbapenems in Gram-negative bacilli (*n* = 43 tests) was concordant in 86.0% (*n* = 37); of those, 36 belonged to *Enterobacterales* and one to *Pseudomonas aeruginosa*; three isolates (*Enterobacter cloacae*-complex and *Klebsiella pneumoniae*) that were tested susceptible to meropenem at CHU-B were resistant upon retesting whereas three *Enterobacterales* isolates that were tested intermediate were susceptible to meropenem upon retesting.

## Discussion

Our study showed that automated BC was superior to manual BC in this resource-limited setting at a University hospital in Côte d'Ivoire. The yield was higher and turnaround times were shorter in automated compared to manual BC. Automated BC enabled the laboratory to inform the clinicians 76 h earlier about first positive BC results than with manual BC. Furthermore, our study increased BC sampling from 24 to 63 BC per month. These findings are in accordance with other studies in resource-limited settings (12, 20). Additionally, the proportion of false-positive BC and therefore the workload could be reduced with automated BC significantly. Before considering the implementation of automated tools in resource-limited settings, the conditions, costs, and needs of automated vs. manual methods should be carefully considered according to the local setting. The proportion of BC with growth of a pathogen was high in both, automated and manual BC, and above the targeted 5-15% (21). This could be an indicator that still too few BC were sampled and patients with blood stream infections were missed. Contamination of BC was high in automated and manual BC (48 and 43%, respectively), although clinical training on good practice BC sampling supported our study. The proportion of contaminated BC was higher in our study than in comparable studies in resource-limited settings and above the targeted 3% (20, 22–24). As most contaminants belonged to the typical flora of the skin, we assume that contamination occurred during sampling due to insufficient disinfection.

We found that half of the results of AST were concordant in automated and manual BC if the same pathogen was found. Differences affected several antibiotic classes. An internal quality control study in Switzerland found that even in high-resource settings 14% of samples were not accurately retested in the same microbiological laboratory and 3% of tested antibiotics were interpreted differently (25). By the time of the study, internal quality controls including sterility tests were in place, but commercially available quality control strains were not established.

For a good quality management system the implementation of external quality control programmes is essential (13, 26). As no external quality control was in place at CHU-B at the time of the study, we retested isolates in a German routine microbiology laboratory. The comparison of manual and automated methods for identification should be interpreted with caution as these use different principles. The identification of microorganisms from CHU-B posed difficulties for the automated tools in retesting and had to be retested several times or could not be identified at all. In some cases, more isolates were recovered in the samples upon retesting. The additionally recovered isolates could have been present but not discriminated at CHU-B or contamination occurred during storage or planting. In only 11%, a different bacterial genus was identified in retesting. This highlights the good quality of manual identification despite the limited resources. Manual identification did not allow discriminating all bacteria to species level, but in resource-limited settings grouping of bacteria according to their clinical relevance might be reasonable and shorten turnaround time (11, 27). No systematic discordance between the identification tools could be identified. The differences might partly be explained by contamination or documentation error. Another reason might be that sheep blood was not available for the preparation of blood agar. Human blood discarded by blood bank had to be used instead. After the study, the laboratory successfully introduced self-prepared sheep blood agar sustainably. This was enabled by an expert exchange with other sub-Saharan countries. Different AST results were found in 23% of tested antibiotics upon retesting although the same interpretation reference was used predominantly. EUCAST had been implemented at CHU-B as this is a freely available reference (17). However, differences could partly be explained by different AST methods and references. If no EUCAST breakpoints for disk diffusion were available, the references from the Clinical and Laboratory Standards Institute (CLSI) were used at CHU-B. This could explain why teicoplanin and vancomycin were interpreted as more resistant at CHU-B than in retesting. Inter-test variability should also be considered to explain some of the differences. The culture of microorganisms is influenced by many factors, e.g., culture media, inoculation of plate, incubation atmosphere and time or stochastic growth variability. We experienced that the provided EUCAST learning materials were very helpful to introduce EUCAST, but in part too sophisticated. Difficulties implementing EUCAST in resource-limited settings is especially hampered because defibrinated horse blood is not available for AST of fastidious bacteria. This has been published also by others and we recommend a low-resource adapted EUCAST version to overcome these obstacles (4, 28).

We asked clinicians to take blood for culture whenever they assumed a bloodstream infection, but we did not assess how well the recommendations were followed. To increase adherence and to avoid overloading of the laboratory capacities, we asked clinicians for one BC pair (automated and manual) only. If multiple BC were collected from one patient, these were counted as multiple cases. Anaerobic sub-culture was not available in the laboratory. For CO2-enriched cultures, a candle jar was used. This might explain why only few streptococci were found. Additionally, the attending microbiologist evaluated if the recovered bacteria were defined as contaminant or pathogen individually based on multiple criteria, not only identification. This might lead to different results.

Our study emphasizes that the implementation of automated tools such as BC incubation might be of value, but that good quality manual microbiological methods for identification and AST might not be inferior to automated processes. Before considering the implementation of automated tools, they should be adapted to the conditions and needs in resource-limited settings as they are costly, require regular training and maintenance and are not adapted to the tropical climate and environment (11). In our study setting, the price for automated BC bottles was higher than for BC with self-prepared BHI broth. However, we could not establish a sustainable delivery of suitable, reusable glass bottles for the increased BC requests. Therefore, we had to purchase commercially available manual BC bottles, so the price did not differ much from the price of automated BC bottles. Moreover, the supply chain for automated BC bottles in our study setting was more reliable. The maintenance and technical support of automated BC is critical. Laboratory staff trained in troubleshooting for automated BC were available in the laboratory throughout our study period. All microbiological laboratory staff were trained in the loading and unloading of automated BC stepwise. The BacT/ALERT^®^ 3D system was placed in a separate and clean room with air-conditioning. We did not experience power shortcuts with breakdown of the machine. For sustainable implementation of automated tools, the system has to be integrated in the laboratory strategy, funding beyond study periods has to be assured and therefore, all levels, including ministries of health, should be integrated in the process.

In expert interviews conducted in Africa and Asia, all interviewers listed environmental conditions as a significant challenge for BC processing (29). Further challenges are the different spectrum of “tropical” bacteria. Our study showed that automated tools might have difficulties identifying these bacteria. More important than implementing automated identification and AST tools are the implementation of good quality management systems including internal and external quality control (27). Laboratories face huge problems as necessary reagents for manual identification are not available anymore. To ensure good quality manual identification it is essential to maintain the production and delivery of these necessary reagents.

Our study was one step to implement and improve diagnostic stewardship at CHU-B. We were able to improve sampling and processing of BC in the laboratory. Diagnostic stewardship should be further strengthened and focus intensively on sampling, specimen transport and reporting of results. Continuous mentorship programmes including technical support and training should be maintained. Our study was an important step to include AST results in the local and national AMR surveillance and enables the comparison of BC results between different sites using either automated or manual BC.

## Conclusion

Automated BC could be a valuable tool for the microbiological laboratory in resource-limited settings if the maintenance is assured and the staff is well-trained. Automated BC was superior to manual BC in sensitivity and timeliness. Mentorship to improve diagnostic stewardship further should focus on BC sampling and reporting of results. It is very important to implement and integrate good laboratory practices to improve manual microbiological methods for identification and AST and to ensure availability of necessary reagents. The implementation of automated tools should be decided individually according to economic considerations, number of samples processed, stable supply chain of consumables, and technical sustainability.

## Data Availability Statement

The datasets presented in this article are not readily available because the data will be made available upon reasonable request to interested researchers. Requests to access the datasets should be directed to laera@rki.de.

## Ethics Statement

The studies involving human participants were reviewed and approved by Charité University Ethics Committee Berlin, Germany (reference number: EA2/230/17) and the National Ethics Committee for Research in Abidjan, Côte d'Ivoire (reference number: 141/MHSP/CNER-km). Written informed consent from the participants' legal guardian/next of kin was not required to participate in this study in accordance with the national legislation and the institutional requirements.

## Author Contributions

AL, MN'G, FT, TE, KN, and CA-K contributed to conception and design of the study. AL, MN'G, FT, and CA-K implemented the study, generated and collected the data in Côte d'Ivoire. RI and JF generated and collected the data in Germany. KN, ST, FL, TE, and CA-K supervised the whole study and analysis. AL, KG, and JF organized the database. AL performed the data analysis and wrote the first draft of the manuscript. All authors critically reviewed the manuscript and approved the submitted version.

## Conflict of Interest

RI and JF were employed by the company MVZ Labor_28 GmbH. The remaining authors declare that the research was conducted in the absence of any commercial or financial relationships that could be construed as a potential conflict of interest.

## Supplementary Material

Supplementary Figure 1 Identification flow chart for manual identification of Gram-positive cocci and Gram-negative bacilli used during the study period, CHU-B, Côte d'Ivoire 2017–2018.
