# Supplementary figures and images for: Implementation of Automated Blood Culture With Quality Assurance in a Resource-Limited Setting
Source: Front Med (Lausanne). 2021 May 21;8:627513. doi: 10.3389/fmed.2021.627513 (PMC8176090; doi:10.3389/fmed.2021.627513)

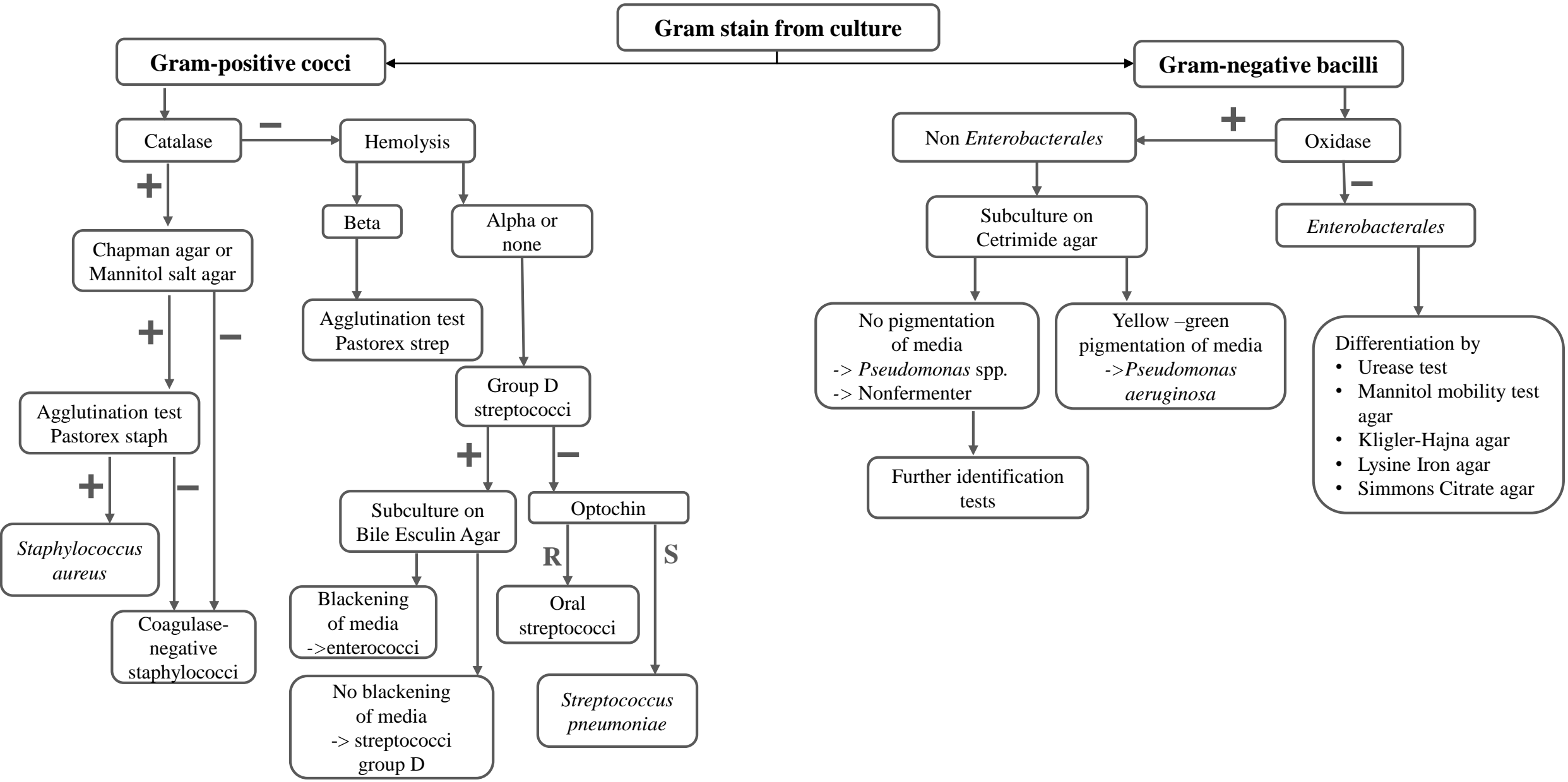

R = resistant, S = susceptible

Supplement: Supplementary Figure 1 — Identification flow chart for manual identification of Gram-positive cocci and Gram-negative bacilli used during the study period, CHU-B, Côte d'Ivoire 2017–2018. [file Data_Sheet_1.pdf]
